# Machine learning algorithms to identify cluster randomized trials from MEDLINE and EMBASE

**DOI:** 10.1186/s13643-022-02082-4

**Published:** 2022-10-25

**Authors:** Ahmed A. Al-Jaishi, Monica Taljaard, Melissa D. Al-Jaishi, Sheikh S. Abdullah, Lehana Thabane, P. J. Devereaux, Stephanie N. Dixon, Amit X. Garg

**Affiliations:** 1 Lawson Health Research Institute, 800 Commissioners Rd E, London, ON Canada; 2 Clinical Epidemiology Program, School of Epidemiology and Public Health, Ottawa Hospital Research Institute, University of Ottawa, 501 Smyth Road, Ottawa, ON Canada; 3 London Health Sciences Centre, 800 Commissioners Rd E, London, ON Canada; 4 Department of Computer Science, Western University, 1151 Richmond St, London, ON Canada; 5 Department of Health Research Methods, Evidence, and Impact, McMaster University, 1280 Main St W, Hamilton, ON Canada

**Keywords:** Cluster randomized controlled trial, Machine learning, Bibliographic databases, Sensitivity and specificity, Prediction

## Abstract

**Background:**

Cluster randomized trials (CRTs) are becoming an increasingly important design. However, authors of CRTs do not always adhere to requirements to explicitly identify the design as cluster randomized in titles and abstracts, making retrieval from bibliographic databases difficult. Machine learning algorithms may improve their identification and retrieval. Therefore, we aimed to develop machine learning algorithms that accurately determine whether a bibliographic citation is a CRT report.

**Methods:**

We trained, internally validated, and externally validated two convolutional neural networks and one support vector machine (SVM) algorithm to predict whether a citation is a CRT report or not. We exclusively used the information in an article citation, including the title, abstract, keywords, and subject headings. The algorithms’ output was a probability from 0 to 1. We assessed algorithm performance using the area under the receiver operating characteristic (AUC) curves. Each algorithm’s performance was evaluated individually and together as an ensemble. We randomly selected 5000 from 87,633 citations to train and internally validate our algorithms. Of the 5000 selected citations, 589 (12%) were confirmed CRT reports. We then externally validated our algorithms on an independent set of 1916 randomized trial citations, with 665 (35%) confirmed CRT reports.

**Results:**

In internal validation, the ensemble algorithm discriminated best for identifying CRT reports with an AUC of 98.6% (95% confidence interval: 97.8%, 99.4%), sensitivity of 97.7% (94.3%, 100%), and specificity of 85.0% (81.8%, 88.1%). In external validation, the ensemble algorithm had an AUC of 97.8% (97.0%, 98.5%), sensitivity of 97.6% (96.4%, 98.6%), and specificity of 78.2% (75.9%, 80.4%)). All three individual algorithms performed well, but less so than the ensemble.

**Conclusions:**

We successfully developed high-performance algorithms that identified whether a citation was a CRT report with high sensitivity and moderately high specificity. We provide open-source software to facilitate the use of our algorithms in practice.

**Supplementary Information:**

The online version contains supplementary material available at 10.1186/s13643-022-02082-4.

## Introduction

Randomized controlled trials (RCTs) provide a robust study design to evaluate health interventions. Compared to individually randomized trials, which randomize individuals, cluster randomized trials (CRTs) allocate groups of people, such as medical practices, hospitals, nursing homes, schools, or even entire communities [1–6]. Cluster randomized trials are often used to test interventions in education, social welfare, and public health.

We and other methodologists increasingly search MEDLINE, EMBASE, and other bibliographic databases for CRT reports [7–10]. Unfortunately, the authors of CRT reports do not always adhere to the Consolidated Standards of Reporting Trial (CONSORT) Statement Extension for Cluster Randomized Trial requirements to explicitly identify the design as “cluster randomized” in the title or abstract of the report [11, 12]. In a review of 162 trials, about half of CRT reports clearly documented the study design in titles or abstracts, about one-quarter can be identified based on the reported units of randomization but are not amenable to electronic searching, and the remaining quarter cannot be identified except through manual inspection of the full-text article [11, 12]. As such, it is challenging to retrieve reports of CRTs from bibliographic databases. As of June 2020, we estimated that less than 0.1% of the 17.5 million citations in PubMed over the prior two decades were CRT reports; finding reports of CRTs in bibliographic databases are a problem akin to screening for rare diseases in the general population [13].

A common practice for identifying CRT reports may involve using an established *database search filter* [12]. Search filters contain combinations of text strings and database tags developed by information specialists. An existing search filter captures over 90% of CRT-related articles [12]. However, this filter also captures many articles that are not CRT reports, and CRT reports represent only 10 to 15% of articles identified by the search filter [12]. Thus, a reviewer needs to screen seven to ten records to identify one CRT report. This process is time-consuming, with a chance of human error.

Machine learning and text mining techniques can extract useful information from an article’s citation (e.g., title and abstract) and have proved successful in classifying whether an article’s citation is an RCT [14–16]. In this study, we developed and internally and externally validated machine learning algorithms to accurately determine whether an article citation is a CRT report so it can be retrieved when searching bibliographic databases. Our machine learning classifier will aid methodologists and systematic reviewers in identifying CRT reports in a fraction of the time compared to the usual screening of all articles.

## Methods

This section is organized into five subsections. First, we describe the data sets used to train, internally validate, and externally validate the machine learning algorithms. Second, we describe the machine learning algorithms. Third, we explain how we processed the data, trained our algorithms, and optimized each algorithm’s hyperparameters. Fourth, we describe how we combined our models (an ensemble method) to boost the algorithm’s predictive performance compared to a single model. Finally, we describe the evaluation metrics used to test our algorithms’ overall performance.

### Datasets

#### Training and internal validation

To identify article citations for our training and internal validation sets, we used a previously published CRT search filter in MEDLINE and EMBASE that yielded 87,633 citations published between January 1, 2000, and December 31, 2019 (see Additional file 1 for additional details about the search) [12]. We randomly selected 5000 citations from these records for training and internal validation. AAA and MDA independently classified whether each citation was a CRT report, and they had over 97% agreement in their classification; discrepancies were resolved through discussion. Inclusion criteria were primary or secondary reports of CRTs, CRT protocols, or pilot and feasibility of CRTs. Citations meeting those eligibility criteria were included regardless of the setting, clinical area, or cluster type. Exclusion criteria were trials reporting only baseline findings, quasi-randomized trials, studies reporting process evaluation or method papers, individually randomized trials, observational studies, editorials, and mechanistic studies. The reviewers based their assessment primarily on the title and abstract, but the article’s full text was reviewed when the unit of randomization was unclear. We expected that 10 to 15% of the 5000 articles would be CRT reports [12].

#### External validation dataset

We evaluated our algorithms’ performance against an external dataset that included 1988 articles. These articles were confirmed primary reports of RCTs, of which 688 were CRT reports and the rest were individually randomized trials. This dataset has been described elsewhere [17]. Briefly, the authors identified pragmatic clinical trials using a sensitivity-maximizing pragmatic search filter; this search filter is independent of this study’s CRT search filter [18]. The search filter for the external dataset was applied in MEDLINE on April 3, 2019, for the period between January 1, 2014, and April 3, 2019.

From the 1988 articles, we removed 72 articles that were captured in the training or validation datasets. We applied this exclusion criterion to avoid data leakage that would artificially inflate the models’ performance. See Additional file 2 for additional details about the external dataset.

### Machine learning algorithms

#### Convolutional neural networks

Although convolutional neural networks were initially developed and used for image classification, they have been used extensively for text classification [19–22]. These models use low-dimensional (typically 50 to 300) continuous vectors to represent words (word embeddings). The convolutional neural network algorithm takes an input text document and assigns weights and biases to different neurons based on extracted features during the learning process. Next, the algorithm passes linear filters represented with corresponding weight vectors over word embeddings. The filters start at the beginning and move sequentially through the document. As such, each filter produces a vector that is proportional in size to the document length. Filter outputs are then combined by extracting the maximum value on each filter output vector (i.e., max-pooling). Finally, the algorithm concatenates these scalar values to form a vector representation of the entire document that becomes the input for the classification prediction layer (Additional file 3: Fig S1). A detailed description of CNNs used for natural language processing is provided by Wang (2019) [23].

#### Support vector machines

Support vector machines identify the best hyperplane separating classes (e.g., CRT vs. non-CRT report) in high-dimensional space [24]. This method uses kernel functions (i.e., a similarity function between a pair of records) that can be a linear, polynomial, sigmoid, or radial basis function. These kernel functions transform the data into the form necessary for prediction. We trained the models using the abovementioned kernel functions and found the radial basis function performed best (see the “Hyperparameter optimization” section). Additional file 4: Fig. S2 provide more details on the two parameters associated with the radial basis function: gamma (kernel coefficient) and c (regularization parameter).

### Data preprocessing, model features, and hyperparameter choices

#### Data preprocessing

We concatenated each citation’s title, abstract, keywords, and subject headings. Next, we conducted several data cleaning steps for each record, including putting text in lowercase, removing brackets/parentheses, punctuations, numbers, and words containing numbers. When a citation had a structured abstract, we removed the discussion and conclusion because these sections rarely contained relevant information about the study design. We then tokenized titles, abstracts, keywords, and subject headings. Finally, we removed stopwords such as *“and,” “the,” “we,” and “was” (i.e.,* common words with low informational content).

#### Word embeddings

For the convolutional neural network models, we used word embeddings as feature parameters. A word embedding is a learned representation for text, where words that have a similar meaning (i.e., used in a similar context) will have a similar representation in vector space (e.g., “mother,” “father,” “parent,” “guardian” would have similar vector representation). An unsupervised neural network maps each word to one vector. We trained two word-embedding models: Word2Vec and FastText, with the skip-gram architecture and ten iterations (Additional file 5) [25, 26]. We trained the word embedding models using the 87,633 articles retrieved by our search strategy.

#### Term frequency-inverse document frequency

We used the term frequency-inverse document frequency (TF-IDF) method to weigh the relative importance of unique words in our dataset for support vector machines [24, 27]. The TF-IDF weights increase proportionally to the number of times a word appears in a document. These weights are offset by the number of records containing that word, which helps to adjust for expressions frequently appearing in the dataset (e.g., the term “random”). Information retrieval, text mining, and user modeling tasks commonly use the TF-IDF method as a weighting factor [28].

#### Handling class imbalance

There are far fewer CRT than non-CRT reports, which poses a problem for standard learning algorithms that maximize predictive accuracy. Given this class imbalance scenario, we observed high model accuracy by uniformly predicting the majority class (i.e., non-CRT reports). We handled class imbalance by (1) constructing a dataset that included all CRT reports but only a random subsample of non-CRT reports and (2) by adjusting class weights where each CRT training example carried more weight than non-CRT reports [29]. Table 1 shows the details of the search space and the chosen sampling ratio.

**Table 1:** Hyperparameter search space for convolutional neural networks and support vector machines

**Hyperparameter**	**Values checked**	**Chosen value**
**For all models**		
Sampling ratio (non-CRT:CRT)	(1411:589), (2411:589), (3411:589), (4411:589)	3411: 589
Class weights (non-CRT:CRT)	(1:1), (1:5), (0.59:3.4), (1:17), (1:20)	0.59: 3.4
Metric	AUROC	AUROC
**Convolutional neural network—Word2Vec**		
Max length of each abstract	100, 150, 200, 250, 300, 350	300
Batch size (distribution)	Uniform distribution (10, 30)	11
Learning rate (distribution)	Uniform distribution (0.0005, 0.005)	0.0047
Dropout rate (distribution)	Uniform distribution (0.1, 0.5)	0.29
Number of filters (distribution)	Uniform distribution (64, 1526)	923
Kernel size (distribution)	Uniform distribution (3, 12)	8
Number of epochs (distribution)	Uniform distribution (3, 20)	7
Constraint applied to the kernel matrix (distribution)	1, 1.5, 2, 2.5, 3	2
Optimizer (distribution)	Adadelta, Adam	Adam
Embedding	Skip-gram; CBOW	Skip-gram
Embedding dimensions	50, 100, 200, 300	100
Number of embedding iterations	5, 10, 15, 20	10
Loss	Binary cross-entropy	Binary cross-entropy
**Convolutional neural network—FastText**		
Max length of each abstract	100, 150, 200, 250, 300, 350	300
Batch size (distribution)	Uniform distribution (10, 30)	16
Learning rate (distribution)	Uniform distribution (0.0005, 0.005)	0.0026
Dropout rate (distribution)	Uniform distribution (0.1, 0.5)	0.47
Number of filters (distribution)	Uniform distribution (64, 1526)	532
Kernel size (distribution)	Uniform distribution (3, 12)	11
Number of epochs (distribution)	Uniform distribution (3, 20)	14
Constraint applied to the kernel matrix (distribution)	1, 1.5, 2, 2.5, 3	2
Optimizer (distribution)	Adadelta, Adam	Adam
Embedding	Skip-gram; CBOW	Skip-gram
Embedding dimensions	50, 100, 200, 300	100
Number of embedding iterations	5, 10, 15, 20	10
Loss	Binary cross-entropy	Binary cross-entropy
**Support vector machines**		
Kernel	linear, polynomial, sigmoid, or radial basis function	Radial basis function
Kernel coefficient	1, 0.1, 0.01, 0.001, 0.0001	0.001
Regularization parameter	1, 10, 100, 1000	100
Ngrams	1, 1 to 2, 1 to 3, 1 to 4	1-gram and bi-gram (1 to 2)
Word Vectorization	Bag of Words, TF-IDF	TF-IDF

*CRT* Cluster randomized trial, *Ngrams* A sequence of *n* words from a text document, *TF-IDF* Term frequency-inverse document frequency, *CBOW* Continuous bag of words model, *AUROC* Area under the receiver operating characteristic curve

#### Hyperparameter optimization

It was impossible to conduct a grid search over all specified hyperparameters for the convolutional neural network models. We used the *hyperopt* python library, which implements Bayesian hyperparameter optimization, to optimize the algorithm’s hyperparameters and achieve the highest algorithm performance [30]. We implemented the Tree of Parzen Estimators (TPE) algorithm with 500 iterations [31, 32]. We also optimized the class weighting, the sampling ratio, and the L1 regularization strength. We examined the effect of different numbers and sizes of filters and differing dropout rates. Dropout rates influence the proportion of neural network connections randomly dropped during training, a strategy used to prevent overfitting [33]. Finally, we examined the effect of varying the vocabulary size where we retained the *N* most common words (e.g., 5000) from the training data. Table 1 shows the full details of the search space and the chosen hyperparameters.

We performed a grid search over all hyperparameters for support vector machines, including sampling ratio, class weights, kernel functions, kernel coefficient(s), regularization parameter, and word vectorization. We also compared the model’s predictive ability when using unigrams, bigrams, and trigrams. Table 1 provides the search spaces and chosen hyperparameters.

### Ensembling

Ensemble learning helps improve results from machine learning models by combining two or more models to boost predictive performance compared to a single model [34]. We evaluated the two convolutional neural networks and support vector machine models individually and as an ensemble of all three algorithms. We estimated the final predicted probability that an article was a CRT report by calculating the average probability score across the three ensembled algorithms.

### Evaluation methods

The algorithms output a probability score from 0 to 1 that an article citation was a CRT report. For each algorithm, we plotted the area under the receiver operating characteristic curve (AUC) as the true positive rate by the false positive rate. The AUC values range between 0 and 1. The AUC value provides information about how well an algorithm can distinguish CRT from a non-CRT report; the closer the AUC value to 1, the better the algorithm predicts non-CRT reports as non-CRT and CRT reports as CRT. The bootstrap procedure in the pROC package in R 3.6.1 was used to estimate the AUCs confidence intervals [35]. As a secondary measure, we estimated the number needed to screen, defined as the average number of algorithm-positive articles manually read to retrieve one CRT report. The prevalence of CRT reports in the respective search strategy and domain area influences the number needed to screen and should be interpreted with caution.

To enable the classification of articles, we chose a threshold probability score to decide whether an article is a CRT report. An article with a probability score greater than the threshold was labeled as a CRT report. Thus, we aimed to choose a probability threshold that would lead to the final algorithm’s sensitivity score greater than 95% for the internal validation dataset without significantly harming the specificity.

The final algorithms were chosen using the best-performing hyperparameters trained on the entire training and internal validation datasets. We then assessed the best-performing algorithms on an external dataset. We conducted data processing and analyses using Python 3.7.7; Additional file 6 describes the python libraries used for this project [36–46].

## Results

**Fig. 1:**
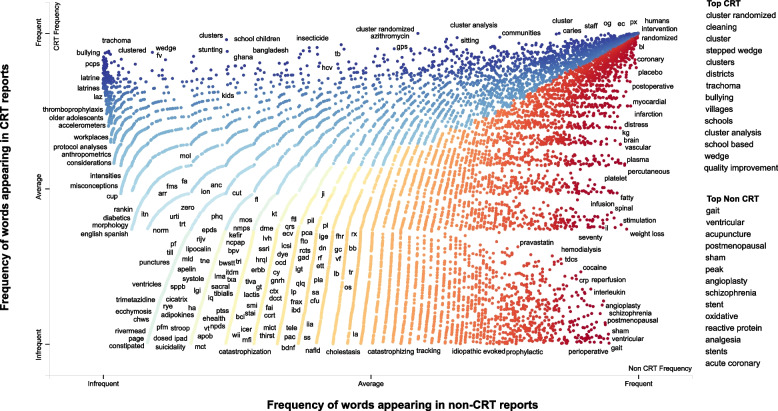
Scatter text visualization of words and phrases used in our dataset. Points are colored blue or red based on related terms with cluster randomized trials (CRT) or non-CRT citations. The dataset consisted of 589 CRT (111,492 words) and 4411 non-CRT citations (816,167 words). The terms associated with each category are under “top CRT” and “top non-CRT” headings. Interactive version of the figure: (interactive Fig. 1  https://mlscreener.s3.ca-central-1.amazonaws.com/Scatter_plot1.html) (*Note: The file size for the interactive figure is large and can take several minutes to load in a browser*)

From the 5000 selected articles, 589 were confirmed to be CRT reports, and the remaining articles were either not CRT reports or were otherwise ineligible. We classified the design for 850 (17%) of the 5000 articles based on the full-text article, while the remaining articles were classified based on the title and abstract alone; we reviewed the full text when the randomization unit was unclear. The 589 CRT citations had 111,492 words, and the 4411 non-CRT citations had 816,167 words. Figure 1 illustrates a scatter plot of terms associated with CRT and non-CRT reports. For example, from the interactive version of Fig. 1 (https://mlscreener.s3.ca-central-1.amazonaws.com/Scatter_plot1.html), 67% of CRT-related articles compared to 1.7% of non-CRT articles contained the term “cluster” in their title, abstract, keyword, or as a subject heading.

**Fig. 2:**
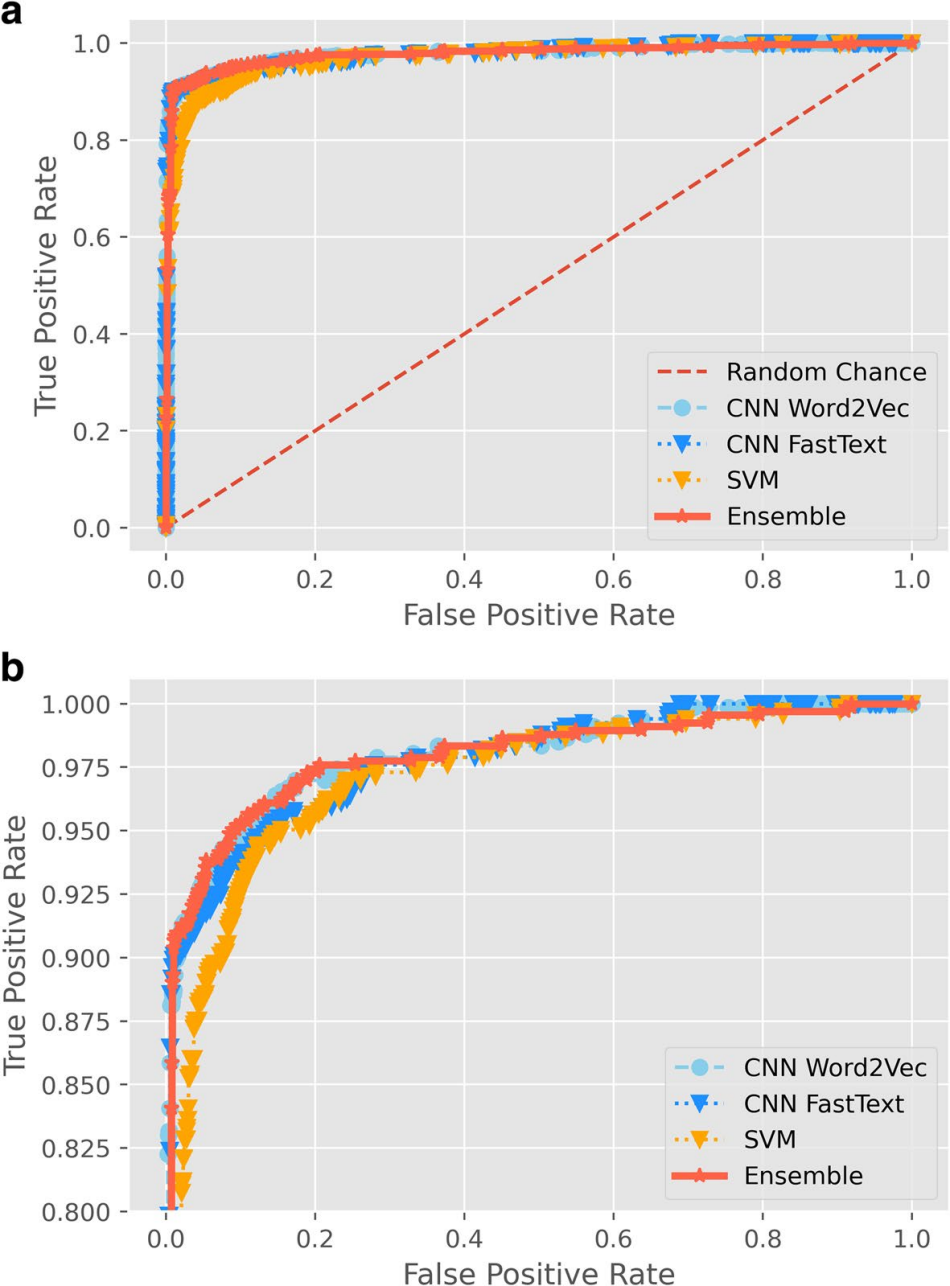
Receiver operating characteristic curves of (1) convolutional neural network using Word2Vec word embedding, (2) convolutional neural network using FastText word embedding, (3) support vector machine (SVM), and (4) ensemble model. **A** A zoomed-out version and **B** zoomed to accentuate variability in the models’ receiver operating characteristic curves

Table 2 displays each algorithm’s performance characteristics for the internal and external datasets. We evaluated the three machine learning algorithms separately and as an ensemble. The individual algorithms operated well. However, the ensemble discriminated best on the validation dataset with an AUC of 98.6% (95% confidence interval: 97.8%, 99.4%), sensitivity of 97.7% (94.3%, 100%), and specificity of 85.0% (81.8%, 88.1%); Fig. 2 shows the algorithm’s performance. For the internal validation dataset, a person would need to screen 6.8 citations, on average, to identify one CRT report. That number dropped to 1.9 citations when using the ensemble algorithm.

**Table 2:** Model metrics for the internal and external validation datasets

**Dataset**	**AUC, %** **(95% CI)**	**True positive rate sensitivity, %** **(95% CI)**	**False positive rate** **1-specificity, %** **(95% CI)**	**Number needed to screen** **(95% CI)**
**Internal validation** **This dataset had 600 articles, with ~ 15% being CRTs** **Number needed to read: 6.8**^**a**^				
*Convolutional neural network—Word2Vec*	98.2 (96.9, 99.5)	96.6 (92.0, 100)	13.9 (10.7, 17.0)	1.8 (1.6, 2.1)
*Convolutional neural network—FastText*	98.4 (97.3, 99.5)	89.8 (83.0, 96.6)	3.5 (2.0, 5.1)	1.2 (1.1, 1.3)
*Support vector machines*	97.2 (95.7, 98.8)	97.7 (94.3, 100)	19.9 (16.4, 23.2)	2.2 (1.9, 2.6)
*Ensemble*	98.6 (97.8, 99.4)	97.7 (94.3, 100)	15.0 (11.9, 18.2)	1.9 (1.7, 2.2)
**External validation** **This dataset had 1916 articles, with ~ 35% being CRTs** **Number needed to read: 2.9**^**a**^				
*Convolutional neural network—Word2Vec*	97.9 (97.2, 98.6)	97.0 (95.6, 98.2)	20.8 (18.5, 23.0)	1.4 (1.3, 1.5)
*Convolutional neural network—FastText*	97.7 (97.0, 98.4)	91.7 (89.8, 93.8)	4.8 (3.7, 6.0)	1.1 (1.1, 1.1)
*Support vector machines*	96.8 (96.0, 97.6)	97.3 (96.1, 98.5)	32.2 (29.7, 34.9)	1.6 (1.6, 1.7)
*Ensemble*	97.8 (97.0, 98.5)	97.6 (96.4, 98.6)	21.8 (19.6, 24.1)	1.4 (1.4, 1.5)

^a^The number needed to read was calculated as one divided by the % of articles that are CRTs

*AUC* Area under the receiver operating characteristic curve, *CI* Confidence interval

For the external dataset (665 CRT reports of 1916 articles), the ensemble algorithm had an AUC of 97.8% (97.0%, 98.5%), sensitivity of 97.6% (96.4%, 98.6%), and specificity of 78.2% (75.9%, 80.4%) (Additional file 7: Fig. S3). The number needed to read dropped from 2.9 to 1.4 citations after implementing the ensemble algorithm.

## Discussion

Bibliographic databases are often searched for CRT reports [7–10]. Unfortunately, the best bibliographic search filter has poor specificity for capturing CRT reports resulting in researchers screening many irrelevant articles [12]. To our knowledge, our paper presents the first tool that can be used to screen CRT reports. Furthermore, we showed that CRT reports could be reliably classified using an ensemble of machine learning algorithms. We expect our algorithms’ performance will improve overtime because (1) we will continue to fine-tune our algorithms as our repository of CRT reports increase and (2) we expect better reporting of the CRT study design as both journals and the CONSORT Statement Extension for Cluster Trials mandate (or recommend) that authors publishing CRT reports include the study design explicitly in the title and abstract [47].

For systematic reviews of CRT reports, our algorithms can lead to a substantial reduction in the number of citations needed to screen with a low probability that CRT reports are excluded (sensitivity greater than 97%.) To facilitate the use of our algorithms in practice, we have made these algorithms available as an easy-to-use open-source software called *MLScreener* (Download Link**—**Additional file 8). The user conducts their database search with clinical terms using their desired search syntax combined with the existing CRT search filter [12]. The user must save the output of retrieved articles from their preferred bibliographic database in a comma-separated values (CSV) file. *MLScreener* takes the search result (in CSV format) as input and generates a probability score as well as a binary classification of whether the record is a CRT report or not. We have two recommendations when using our MLScreener tool: (1) researchers should consider sorting the classified records based on probability scores in descending order and make an informed decision to stop screening based on a low likelihood of seeing CRT reports in the remaining articles or when resources (time or money) have been spent, and (2) researchers should randomly screen at least 100 records from their list of articles (as an external validation) to ensure the MLScreener tool worked well in identifying CRT reports in their search domain.

As an illustration of our algorithms’ application, we implemented our algorithms on a search strategy that identified articles for a systematic review of CRT reports to capture ethical and methodological reporting issues in the dialysis setting [48]. The search strategy for this review identified 882 potentially relevant articles. Our ensemble algorithm correctly identified 33 of the 34 CRT-related articles that two independent screeners identified in their review; see Additional file 9 for more details. As a result, the number of records required for screening was reduced by more than half (note, this will differ depending on the prevalence of CRT reports in the relevant area).

We estimate that it would require an average of 2 min to screen and assess a single article’s eligibility per reviewer for systematic reviews. In our application dataset (882 records), screening time required approximately 30 h per reviewer, which would have been reduced to 15 h per reviewer if we used our algorithms.

### Error analyses

We conducted error analyses to understand why our models make classification errors. We identified titles/abstracts with higher error rates and diagnosed these errors’ root causes when appropriate. Our models generally had very high sensitivity and infrequently classified CRT reports as non-CRTs. However, when CRT reports were incorrectly classified, the title and abstract generally had little indication about the CRT study design (Additional file 10A). As a result, our screeners often had to review the full-text article to classify these reports.

In contrast, it was common for our models to incorrectly classify a non-CRT as a CRT report (Additional file 10B). This incorrect classification often occurred when the title or abstract included similar text to CRT reports. For example, the word “intervention” near the word “community” or “school-based/classes” near the word “random.” It is challenging to engineer features for such reports without overfitting our models. However, we anticipate these errors will decrease as we fine-tune our models with more data.

### Limitations

First, in the absence of available databases of confirmed CRT reports, we created our training and internal validation datasets from a random sample of 5000 articles published between 2000 and 2019. We did not extract whether the study used an intention-to-treat approach for their primary analysis and did not review the full text of all 5000 articles to judge whether they were a report of a CRT. Thus, we may have missed some CRT reports. However, it is unlikely we missed a significant proportion of articles given our algorithms’ high discriminative ability on the external validation dataset, where the full-text articles were reviewed. Second, articles included in our external dataset were published between 2014 and 2019. Thus, we had no external validation for the period before 2014. However, we expect the reliability of classifying CRT reports to resemble the results reported here. Third, we did not explore Bidirectional Encoder Representations from Transformers (BERT) for this project; BERT has been shown to provide state-of-the-art results for natural language processing tasks [49, 50]. However, the use of transformers may be of interest for future classification projects, for example, classifying whether an RCT is a pragmatic trial. Finally, we did not conduct any user analysis for the proposed *MLScreener* software. Thus, we are unaware of how users will engage and interact with our application.

## Supplementary Information

**Additional file 1. **Justification for using a CRT search filter.

**Additional file 2. **Additional details for the external dataset.

**Additional file 3: Fig. S1.** A general architecture for a convolutional neural work used for text classification.

**Additional file 4: **Description of the gamma and c parameters. **Fig. S2.** This figure shows the decision boundaries and support vectors for different settings of the regularization parameter (C) and Kernel coefficient (gamma). We used the mglearn package to create this figure [27].

**Additional file 5. **Continuous skip-gram architecture.

**Additional file 6. **Python libraries used for this project.

**Additional file 7: Fig. S3.** Probability plot for the CRTs in the first external data classified as a CRT (Figure A, 665 CRTs) and non-CRTs classified as a CRT (Figure B, 1251 non-CRTs). The x-axis depicts the stacked ensemble model's prediction of the article being classified as a CRT. The y-axis represents the proportion of all documents that had the corresponding probability.

**Additional file 8. **Screenshots of the front-end tool used for our machine learning algorithm.

**Additional file 9. **Details and results for applying our model on a dialysis-related dataset. **Table S1.** Number of relevant articles retrieved with and without machine learning algorithm using the demonstration dataset (*n*=882 records). The research objective was to review CRTs in the hemodialysis setting to report key methodological and ethical issues.

**Additional file 10. **Examples of titles and abstracts that were classified incorrectly by our algortihms.

## Data Availability

The training and validation data underlying the results presented in the study are available from: https://github.com/aaljaish/MLScreener.
