# Supplementary material for: Machine learning algorithms to identify cluster randomized trials from MEDLINE and EMBASE
Source: Syst Rev. 2022 Oct 25;11:229. doi: 10.1186/s13643-022-02082-4 (PMC9594883; doi:10.1186/s13643-022-02082-4)
Supplement: Supplementary file 1 — Additional file 1. Justification for using a CRT search filter. [file 13643_2022_2082_MOESM1_ESM.docx]

**Additional file 1**: Justification for using a CRT search filter.

We created a dataset of CRT and non-CRT articles to train and internally validate our machine learning algorithms. Given that the prevalence of CRTs is less than 0.1% in bibliographic databases, we would have needed to screen at least 500,000 articles to identify 500 CRT articles, an inefficient and cumbersome process. Therefore, we used a CRT search filter to increase the prevalence of relevant articles retrieved in our bibliographic search, where we expected about 10% to 15% of articles would be CRTs [1].

Search syntax to identify articles in Medline between Jan 1^st^, 2000 and Dec 31^st^, 2019 in EMBASE Classic+ EMBASE, Ovid MEDLINE(R) Epub Ahead of Print, In-Process & Other Non-Indexed Citations, Ovid MEDLINE(R) Daily, and Ovid MEDLINE(R).

| **Search No.** | **Search** | **Records** |
| --- | --- | --- |
| 1 | randomized controlled trial.pt. | 525702 |
| 2 | animals/ | 8037797 |
| 3 | humans/ | 32495688 |
| 4 | 2 not (2 and 3) | 5739131 |
| 5 | 1 not 4 | 514156 |
| 6 | (cluster$ adj2 randomi$).tw. | 30283 |
| 7 | ((communit$ adj2 intervention$) or (communit$ adj2 randomi$)).tw. | 20137 |
| 8 | group$ randomi$.tw. | 8732 |
| 9 | 6 or 7 or 8 | 57622 |
| 10 | intervention?.tw. | 2425049 |
| 11 | cluster analysis/ | 122853 |
| 12 | health promotion/ | 176542 |
| 13 | program evaluation/ | 80902 |
| 14 | health education/ | 159941 |
| 15 | 10 or 11 or 12 or 13 or 14 | 2837625 |
| 16 | 9 or 15 | 2848506 |
| 17 | 16 and 5 | 107591 |
| 18 | limit 17 to yr="2000 - 2019" | 87633 |

**References**

[1] Taljaard M, McGowan J, Grimshaw JM, Brehaut JC, McRae A, Eccles MP, et al. Electronic search strategies to identify reports of cluster randomized trials in MEDLINE: low precision will improve with adherence to reporting standards. BMC Med Res Methodol. 2010;10:15
