# Supplementary material for: Machine learning algorithms to identify cluster randomized trials from MEDLINE and EMBASE
Source: Syst Rev. 2022 Oct 25;11:229. doi: 10.1186/s13643-022-02082-4 (PMC9594883; doi:10.1186/s13643-022-02082-4)
Supplement: Supplementary file 2 — Additional file 2. Additional details for the external dataset. [file 13643_2022_2082_MOESM2_ESM.docx]

**Additional file 2**: Additional details for the external dataset

We evaluated our algorithms' performance against an external dataset that included 1988 articles. These articles were confirmed primary reports of RCTs, of which 688 were CRT reports and the rest were individually randomized trials. This dataset has been described elsewhere [1]. Briefly, the authors identified pragmatic clinical trials using a sensitivity-maximizing pragmatic search filter; this search filter is independent of this study's CRT search filter [2]. The search filter for the external dataset was applied in MEDLINE on April 3^rd^, 2019, for the period between January 1^st^, 2014, and April 3^rd^, 2019. The 2014 date was chosen because this was the first date the National Library of Medicine began indexing pragmatic clinical trials as a publication type. The authors identified 4337 pragmatic articles from the search strategy, of which we used 1988 trials that were registered in ClinicalTrials.gov. From these 1988 articles, we removed 72 articles that were captured in the training or validation datasets. We applied this exclusion criterion to avoid data leakage that would artificially inflate the models' performance.

**References**

[1] Nicholls SG, Carroll K, Hey SP, et al. A review of pragmatic trials found a high degree of diversity in design and scope, deficiencies in reporting and trial registry data, and poor indexing. J Clin Epidemiol. 2021;137:45‑57.

[2] Taljaard M, McDonald S, Nicholls SG, Carroll K, Hey SP, Grimshaw JM, et al. A search filter to identify pragmatic trials in MEDLINE was highly specific but lacked sensitivity. J Clin Epidemiol. 2020;124:75–84. Elsevier USA.
