# Supplementary material for: Machine learning algorithms to identify cluster randomized trials from MEDLINE and EMBASE
Source: Syst Rev. 2022 Oct 25;11:229. doi: 10.1186/s13643-022-02082-4 (PMC9594883; doi:10.1186/s13643-022-02082-4)

**Additional file 3: Fig. S1**. A general architecture for a convolutional neural work used for text classification.


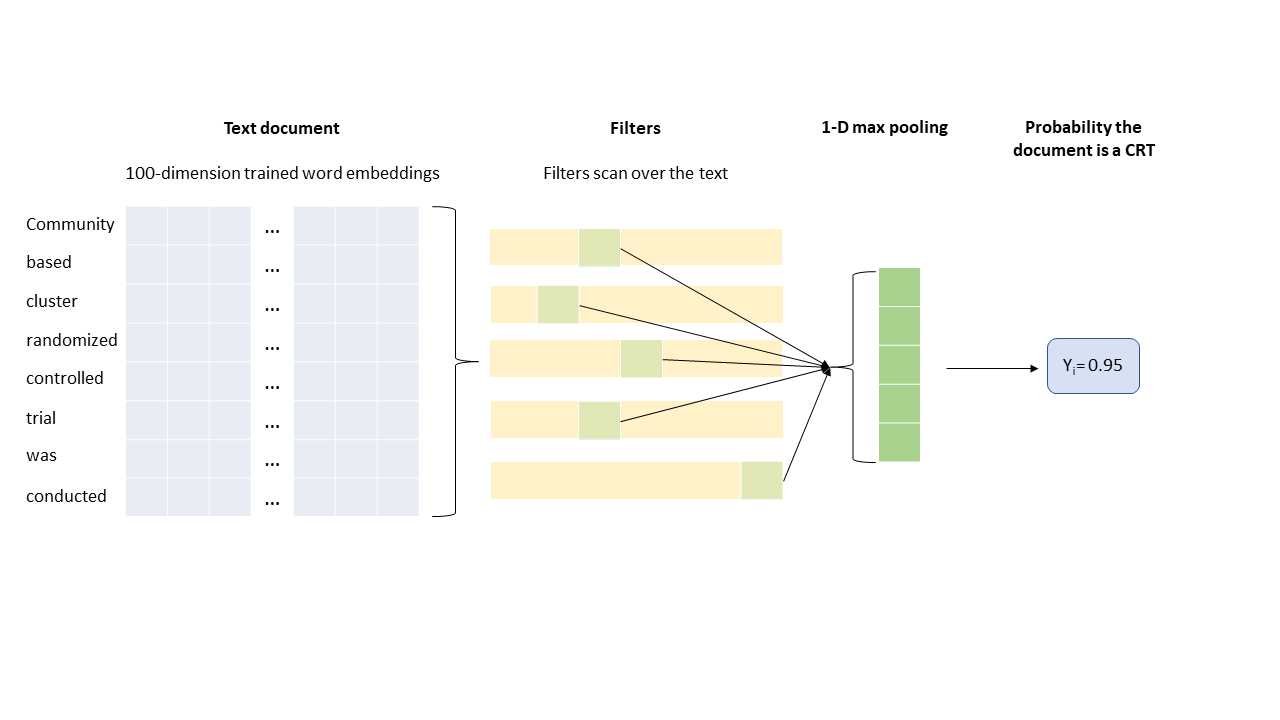

Supplement: Supplementary file 3 — Additional file 3: Fig. S1. A general architecture for a convolutional neural work used for text classification. [file 13643_2022_2082_MOESM3_ESM.docx]
