# Supplementary material for: Machine learning algorithms to identify cluster randomized trials from MEDLINE and EMBASE
Source: Syst Rev. 2022 Oct 25;11:229. doi: 10.1186/s13643-022-02082-4 (PMC9594883; doi:10.1186/s13643-022-02082-4)
Supplement: Supplementary file 4 — Additional file 4: Description of the gamma and c parameters. Fig. S2. This figure shows the decision boundaries and support vectors for different settings of the regularization parameter (C) and Kernel coefficient (gamma). We used the mglearn package to create this figure [27]. [file 13643_2022_2082_MOESM4_ESM.docx]

**Additional file 4**: Description of the gamma and c parameters

The gamma parameter is a cut-off parameter for the Gaussian sphere; increasing gamma increases the training samples' reach and a softer decision boundary (**Fig. S2**) [1]. The c parameter controls the penalty for misclassification. Large values of c correspond to a more substantial error penalty imposed on the model for misclassifying a record ([**Fig.**](#eFigure2_2) **S2**) [1].

**Fig. S2**: This figure shows the decision boundaries and support vectors for different settings of the regularization parameter (C) and Kernel coefficient (gamma). We used the mglearn package to create this figure [2].


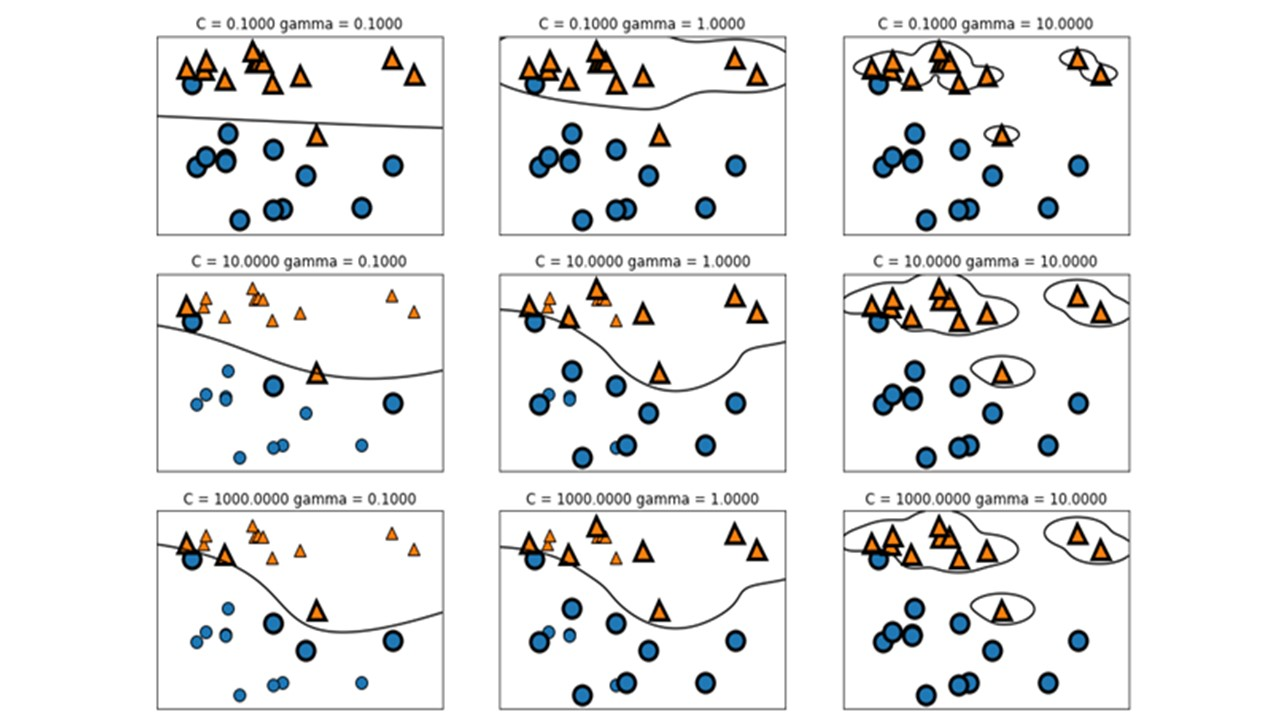


**References**

[1] Raschka S. Python Machine Learning. Hussain A, Youe R, Rajani M, Tuljapurkar R, Chindarkar MS, Khan T, et al., editors. Birmingham: Packt Publishing Ltd; 2015.

[2] Müller AC, Guido S. Introduction to with Python Learning Machine. (Schanafelt D, ed.). O’Reilly Media, Inc.; 2017
