# Supplementary material for: Machine learning algorithms to identify cluster randomized trials from MEDLINE and EMBASE
Source: Syst Rev. 2022 Oct 25;11:229. doi: 10.1186/s13643-022-02082-4 (PMC9594883; doi:10.1186/s13643-022-02082-4)
Supplement: Supplementary file 5 — Additional file 5. Continuous skip-gram architecture. [file 13643_2022_2082_MOESM5_ESM.docx]

**Additional file 5**: Continuous skip-gram architecture

In the continuous skip-gram architecture, the model uses the current word to predict the surrounding window of context words; we set the window to 5 terms in our setting. The skip-gram architecture weighs nearby context words more heavily than more distant context words.
