# Supplementary material for: Machine learning algorithms to identify cluster randomized trials from MEDLINE and EMBASE
Source: Syst Rev. 2022 Oct 25;11:229. doi: 10.1186/s13643-022-02082-4 (PMC9594883; doi:10.1186/s13643-022-02082-4)
Supplement: Supplementary file 6 — Additional file 6. Python libraries used for this project. [file 13643_2022_2082_MOESM6_ESM.docx]

**Additional file 6**: Python libraries used for this project.

1. *pandas* (version 0.25.1) is a package that is intended for working with relational data tables.
2. *NumPy* (version 1.18.1) is a library for scientific computing, which can contain n-dimensional array objects.
3. *sklearn* (version 0.22.1) is a machine learning library for Python. It features various machine learning algorithms like the support vector machine used in this project.
4. *re* is a regular expression library.
5. *string* is a library with a collection of string constants.
6. *nltk* (version 3.4.5) is a Natural Language Toolkit (NLTK) library used for natural language processing.
7. *pickle* library helps create a portable serialized representation of Python objects (e.g., a data frame).
8. *gensim* (version 3.4.0) is a library often used for natural language processing and information retrieval.
9. *keras* (version 2.2.4-tf) is a deep learning framework for developing and evaluating deep learning models.
10. *math* is a module that provides access to mathematical functions.
11. *tensorflow* (version 2.0.0) is a library used for fast numerical computing.
12. *Scattertext* (0.0.2.28) is a package that lets you interactively visualize how two categories of text are different from each other.
13. *matplotlib* (version 3.2.1) is an object-oriented plotting library.
14. *hyperopt* (version 0.2.3) is a Bayesian optimization library that allows for the automatic search of data preparation methods, machine learning algorithms, and model hyperparameters for classification and regression tasks.
