# Supplementary material for: Machine learning algorithms to identify cluster randomized trials from MEDLINE and EMBASE
Source: Syst Rev. 2022 Oct 25;11:229. doi: 10.1186/s13643-022-02082-4 (PMC9594883; doi:10.1186/s13643-022-02082-4)

**Additional file 7: Fig. S3**. Probability plot for the CRTs in the first external data classified as a CRT (Figure A, 665 CRTs) and non-CRTs classified as a CRT (Figure B, 1251 non-CRTs). The x-axis depicts the stacked ensemble model's prediction of the article being classified as a CRT. The y-axis represents the proportion of all documents that had the corresponding probability.


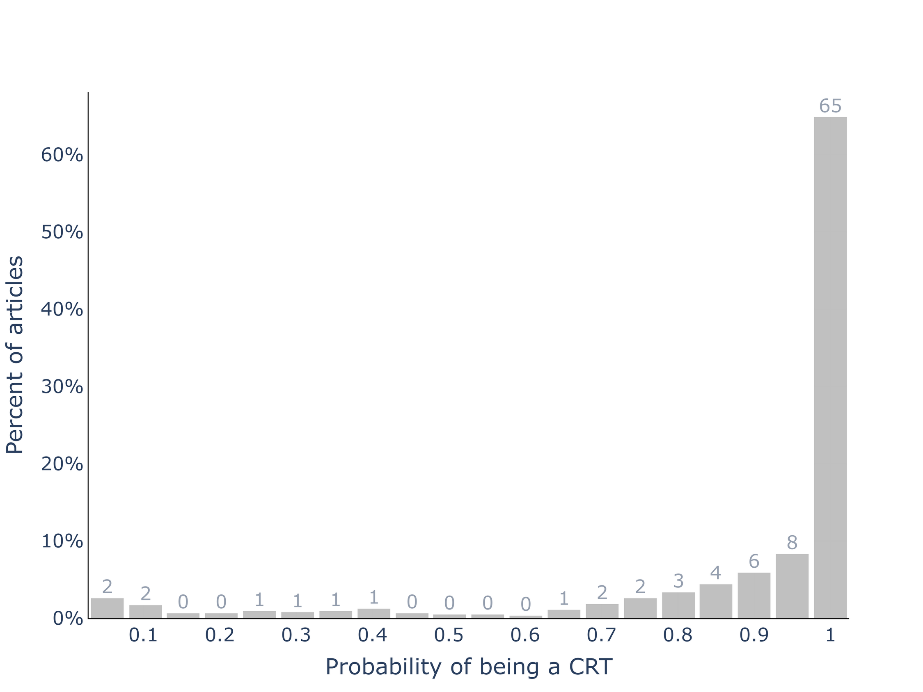


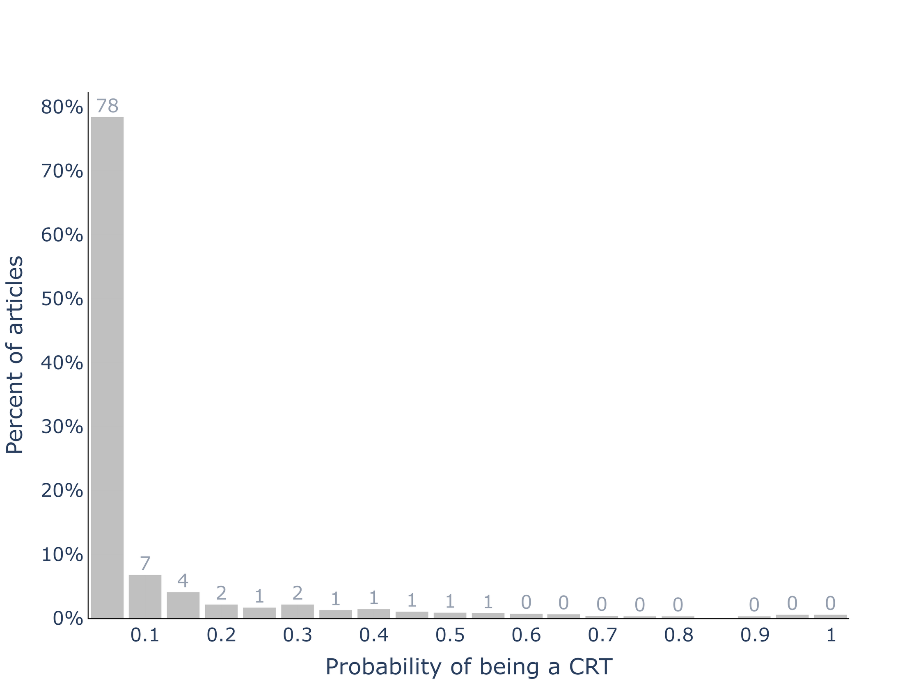

Supplement: Supplementary file 7 — Additional file 7: Fig. S3. Probability plot for the CRTs in the first external data classified as a CRT (Figure A, 665 CRTs) and non-CRTs classified as a CRT (Figure B, 1251 non-CRTs). The x-axis depicts the stacked ensemble model's prediction of the article being classified as a CRT. The y-axis represents the proportion of all documents that had the corresponding probability. [file 13643_2022_2082_MOESM7_ESM.docx]
