# Supplementary material for: Machine learning algorithms to identify cluster randomized trials from MEDLINE and EMBASE
Source: Syst Rev. 2022 Oct 25;11:229. doi: 10.1186/s13643-022-02082-4 (PMC9594883; doi:10.1186/s13643-022-02082-4)

**Additional file 8**: Screenshots of the front-end tool used for our machine learning algorithm.

1. MLScreener Software can be downloaded on any **Windows** machine from: <https://mlscreener.s3.ca-central-1.amazonaws.com/MLScreener.zip>
2. The link above will download a zipped file called “MLScreener.zip”
3. Once downloaded, please right-click on the file and click “Extract All…” on the menu
4. Extract the zipped files in a directory of your choosing
5. From the extracted files, locate the file called “00_MLScreener.exe”
6. Right-click on “00_MLScreener.exe” and click on “Create shortcut”
7. Save the shortcut file in a directory of your choosing
8. Double click on the “Shortcut” 00_MLScreener.exe file to open the application (images below) and follow the instructions in the application.


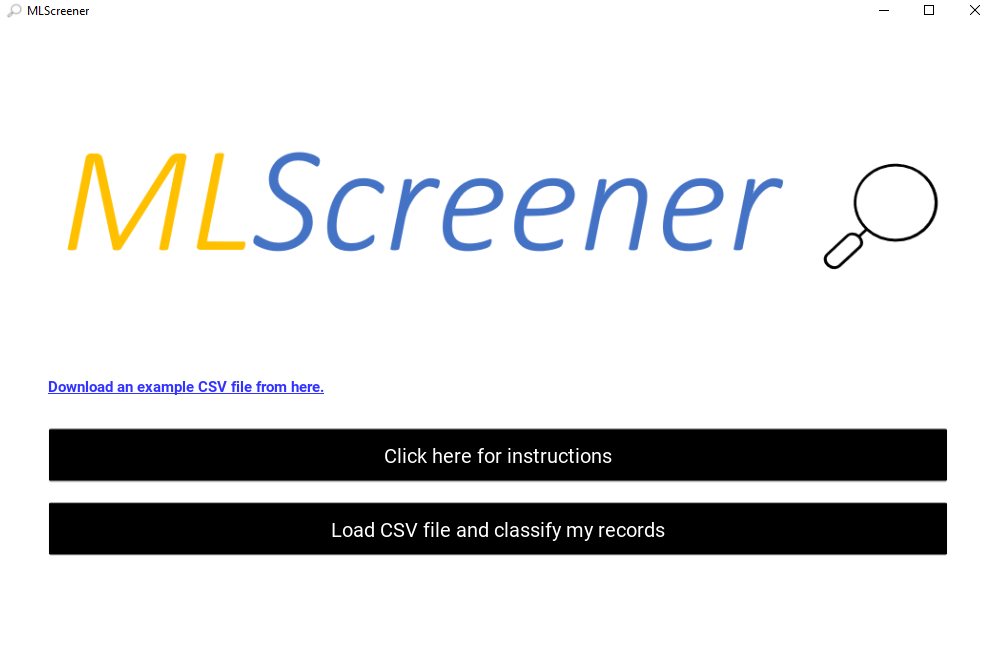


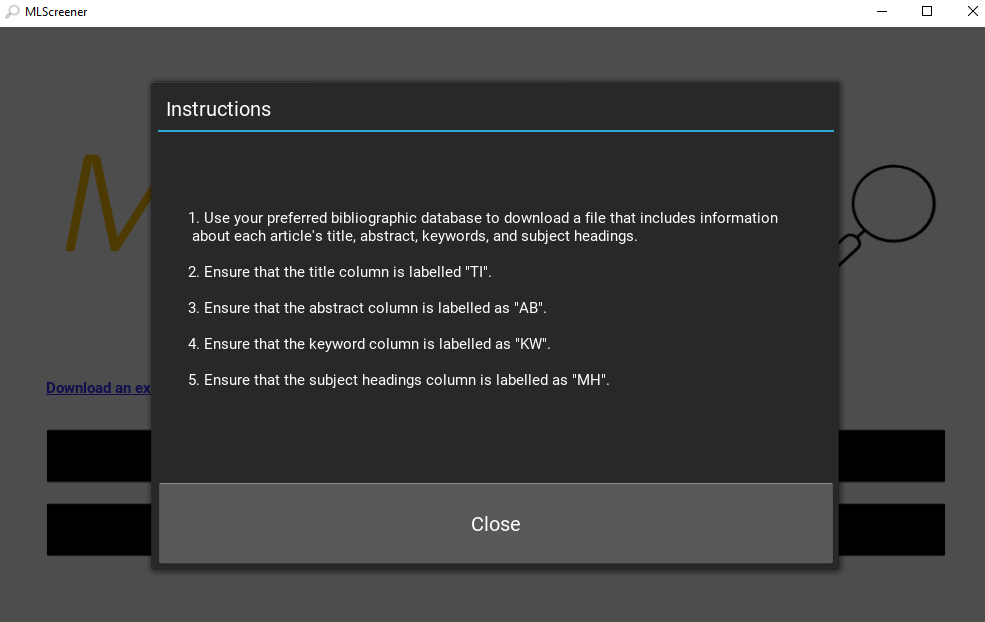

Supplement: Supplementary file 8 — Additional file 8. Screenshots of the front-end tool used for our machine learning algorithm. [file 13643_2022_2082_MOESM8_ESM.docx]
