# Supplementary material for: Machine learning algorithms to identify cluster randomized trials from MEDLINE and EMBASE
Source: Syst Rev. 2022 Oct 25;11:229. doi: 10.1186/s13643-022-02082-4 (PMC9594883; doi:10.1186/s13643-022-02082-4)
Supplement: Supplementary file 9 — Additional file 9. Details and results for applying our model on a dialysis-related dataset. Table S1. Number of relevant articles retrieved with and without machine learning algorithm using the demonstration dataset (n=882 records). The research objective was to review CRTs in the hemodialysis setting to report key methodological and ethical issues. [file 13643_2022_2082_MOESM9_ESM.docx]

**Additional file 9**: Details and results for applying our model on a dialysis-related dataset.

This dataset included 882 articles from a search strategy that combined two search filters to identify CRT reports and dialysis studies [1,2]. Two reviewers screened titles and abstracts to identify CRT reports in the hemodialysis setting. We excluded CRT reports unrelated to in-center hemodialysis. We applied our algorithms to this dataset to evaluate whether it reduced the number of articles needed to screen without excluding hemodialysis-related CRT reports.

The [**Table**](#eTable) **S1** below showed the results when our model was applied in practice to classify CRT reports in the dialysis setting. The models correctly classified 33 of the 34 dialysis-related CRT reports. The number of articles needed to screen to capture a single CRT was reduced from 26 to 11.4.

**Table S1**: Number of relevant articles retrieved with and without machine learning algorithm using the demonstration dataset (n=882 records). The research objective was to review CRTs in the hemodialysis setting to report key methodological and ethical issues.

|  | Number of relevant CRT articles identified | Number of non-relevant articles captured |
| --- | --- | --- |
| Manual screening | 34* | 848 ^++^ |
| Ensemble | 33** | 343 ^++^ |

* There was a total of 36 cluster randomized trials (CRTs) conducted in the hemodialysis setting. Two of the 36 CRT articles were identified in the included articles' reference list and are not included above. The title and abstract of these two had no indication they utilized a CRT study design and these articles were not picked up by the CRT search filter[3,4].

** The missed article stated it was a "group randomized trial" but later stated, "Patients at participating dialysis centers were randomized…[5].

++ A proportion of these articles were CRTs unrelated to the hemodialysis setting and were not relevant to the respective review's research objective.**References**

[1] Taljaard M, McGowan J, Grimshaw JM, Brehaut JC, McRae A, Eccles MP, et al. Electronic search strategies to identify reports of cluster randomized trials in MEDLINE: low precision will improve with adherence to reporting standards. BMC Med Res Methodol. 2010;10:15

[2] Iansavichus AV, Haynes RB, Lee CWC, Wilczynski NL, McKibbon A, Shariff SZ, et al. Dialysis search filters for PubMed, Ovid MEDLINE, and Embase databases. Clin J Am Soc Nephrol. 2012;7:1624–3161

[3] Kauric-Klein Z. Improving blood pressure control in end stage renal disease through a supportive educative nursing intervention. Nephrol Nurs J. 2012;39:217–28.

[4] Howren MB, Kellerman QD, Hillis SL, Cvengros J, Lawton W, Christensen AJ. Effect of a behavioral self-regulation intervention on patient adherence to fluid-intake restrictions in hemodialysis: a randomized controlled trial. Ann Behav Med. 2016;50:167–76.

[5] Waterman AD, Peipert JD. An explore transplant group randomized controlled education trial to increase dialysis patients’ decision-making and pursuit of transplantation. Prog Transplant. 2018;28:174–83.
