# Supplementary material for: Machine learning algorithms to identify cluster randomized trials from MEDLINE and EMBASE
Source: Syst Rev. 2022 Oct 25;11:229. doi: 10.1186/s13643-022-02082-4 (PMC9594883; doi:10.1186/s13643-022-02082-4)
Supplement: Supplementary file 10 — Additional file 10. Examples of titles and abstracts that were classified incorrectly by our algortihms. [file 13643_2022_2082_MOESM10_ESM.docx]

**Additional file 10**: Examples of titles and abstracts that were classified incorrectly by our algortihms.

1. **Example 1**

***True classification:*** Not a CRT report

***Probability of being a cluster trial report:*** 52%

***Title:*** Why is a treatment aimed at psychosocial factors not effective in patients with (sub)acute low back pain?.

***Abstract:***

Psychosocial factors have been shown to play an important role in the development of chronic low back pain (LBP). In our recently completed cluster-randomized trial we found, however, no evidence of an effect of our minimal intervention strategy (MIS) aimed at psychosocial factors, over usual care (UC) in patients with (sub)acute LBP. To explore the reasons why, this paper presents an evaluation of the processes presumably underlying the effectiveness of MIS. General practitioner (GP) attitude was evaluated by the Pain Attitudes and Beliefs Scale and two additional questions. GP behaviour was evaluated by analysing treatment registration forms and patients' responses to items regarding treatment content. Patients also scored items on satisfaction and compliance. Modification of psychosocial measures was evaluated by analysing changes after 6 and 52 weeks on the Fear Avoidance and Beliefs Questionnaire, the Coping Strategies Questionnaire and the 4-Dimensional Symptom Questionnaire. A total of 60 GPs and 314 patients participated in the study. GPs in the MIS-group adopted a less biomedical orientated attitude than in the UC-group, but were only moderately successful in identification of psychosocial factors. Treatment contents as perceived by the patient and patient satisfaction differed significantly between both groups. Changes on psychosocial measures, however, did not differ between groups. The suboptimal identification of psychosocial factors in the MIS-group and the absence of a relevant impact on psychosocial factors may explain why MIS was not more effective than UC.

**Example 2**

***True classification:*** Not a CRT report

***Probability of being a cluster trial report: 34%***

***Title:*** Summer jobs reduce violence among disadvantaged youth.

***Abstract:*** Every day, acts of violence injure more than 6000 people in the United States. Despite decades of social science arguing that joblessness among disadvantaged youth is a key cause of violent offending, programs to remedy youth unemployment do not consistently reduce delinquency. This study tests whether summer jobs, which shift focus from remediation to prevention, can reduce crime. In a randomized controlled trial among 1634 disadvantaged high school youth in Chicago, assignment to a summer jobs program decreases violence by 43% over 16 months (3.95 fewer violent-crime arrests per 100 youth). The decline occurs largely after the 8-week intervention ends. The results suggest the promise of using low-cost, well-targeted programs to generate meaningful behavioral change, even with a problem as complex as youth violence. Copyright © 2014, American Association for the Advancement of Science.

**Example 3**

***True classification:*** Not a CRT report

***Probability of being a cluster trial report:*** 54%

***Title:*** The Chinese community smoking cessation project: a community sensitive intervention trial.

***Abstract:*** This paper describes (1) the design, methods and baseline data of the first smoking cessation clinical trial for Chinese Americans with medical conditions - Chinese Community Smoking Cessation Project (CCSCP); (2) the collaborative process between researchers and the Chinese community; and (3) the barriers and facilitators of implementing the study. CCSCP was a culturally tailored, randomized, smoking cessation trial testing the efficacy of an intensive (physician advice, in-person counseling with nicotine replacement therapy, 5 telephone calls) compared to a minimal (physician advice and self-help manual) intervention. The study applied a community-sensitive research method involving community members in all phases of the research process in San Francisco Bay Area during 2001-2007. CCSCP recruited 464 smokers from health care facilities (79%) located in Chinese neighborhoods and through Chinese language media (21%). Baseline assessments and interventions were conducted in-person using translated and tested questionnaire and intervention materials. The majority of the participants were men (91%) with a mean age of 58.3 years, foreign born (98%), with less than high school education (58%), spoke no English (42%) and in non-skilled or semi-skilled occupations (60%) with <$20,000 household income (51%). Participants smoked regularly on an average 38.6 +/- 17 years, smoked 9.1 +/- 8 cigarettes per day and 85% smoked daily. Cultural tailoring of recruitment methods and intervention design led to successful enrollment and retention of participants, overcoming barriers faced by the participants. Community sensitive collaborative process facilitated implementation of study protocol in community health care settings.

1. **Example 4**

***True classification:*** CRT Report

***Probability of being a cluster trial report:*** 2%

***Title:*** Effect of fluoride-releasing resin composite in white spot lesions prevention: a single-centre, split-mouth, randomized controlled trial.

***Abstract:***

Introduction: The objective of this two-arm split-mouth randomized trial, was to evaluate the ability of fluoride-releasing resin composite to prevent demineralization and white spot lesion (WSL) formation, during orthodontic treatment with fixed appliances.

Methods: Patients needing comprehensive orthodontic treatment were randomly allocated into two groups, according to the half split-mouth technique. This trial examined a total of 300 teeth in each group: the control group, in which brackets were fixed with a non-fluoride-containing adhesive resin; and the intervention group, in which brackets were fixed with a fluoride-containing adhesive resin. Eligibility criteria included Class I malocclusion in the permanent dentition, adequate oral hygiene and no missing teeth, active caries, enamel demineralization, fluorosis staining, or heavy restorations. The primary outcome was the formation of WSLs. Randomization was achieved using a computer-generated random number table; blinding of the patients, assessor, orthodontist and data analysist were achieved. The patients were followed for twelve months, during which time their teeth were checked every three months. To investigate the differences in frequencies and ranks of demineralization and WSL formation between the two groups, odds ratios were computed using mixed modelling (to compensate for the clustered nature of the data) with intervention as a fixed effect and patient as a random effect.

Results: Thirty-four patients (ages, 13-25 years; mean age, 17.6) were randomized into a 1:1 ratio, though four patients dropped out before the start of the treatment. The percentage of the teeth showing the effects of demineralization and WSL formation, increased from 6.3% to 15% for the control group after three and twelve months, respectively, and from 3% to 16.3% for the study group, after three to twelve months, respectively. There were no significant differences between the two groups and no interaction between time and treatment group in the visual inspections (OR 0.79; 95% CI 0.52, 1.21), in DIAGNOdent examinations (OR 0.68; 95% CI 0.43, 1.06), or in photographic images (OR 0.72; 95% CI 0.46, 1.11). No serious harm was observed during the trial.

Limitations: This trial was a single-centre trial, and treatment was carried out by one orthodontist.

Conclusions: Fluoride-containing resin adhesive does not have the desired preventive effect to prevent demineralization and WSL formation, during orthodontic treatment with fixed appliance.

**Example 5**

***True classification:*** CRT Report

***Probability of being a cluster trial report:*** 2%

***Title:*** The role of friends' disruptive behavior in the development of children's tobacco experimentation: results from a preventive intervention study.

***Abstract:***

Having friends who engage in disruptive behavior in childhood may be a risk factor for childhood tobacco experimentation. This study tested the role of friends' disruptive behavior as a mediator of the effects of a classroom based intervention on children's tobacco experimentation. 433 Children (52% males) were randomly assigned to the Good Behavior Game (GBG) intervention, a universal preventive intervention targeting disruptive behavior, and facilitating positive prosocial peer interactions. Friends' disruptive behavior was assessed from age 7-10 years. Participants ' experimentation with tobacco was assessed annually from age 10-13. Reduced rates in tobacco experimentation and friends' disruptive behavior were found among GBG children, as compared to controls. Support for friends' disruptive behavior as a mediator in the link between intervention status and tobacco experimentation was found. These results remained after controlling for friends' and parental smoking status, and child ADHD symptoms. The results support the role of friends' disruptive behavior in preadolescents' tobacco experimentation.

**Example 6**

***True classification:*** CRT Report

***Probability of being a cluster trial report:*** 3%

***Title:*** The impact of an education intervention to change nurses' HIV-related knowledge and attitudes in Lithuania: a randomized controlled trial.

***Abstract:***

The purpose of this study was to describe the effect of an intervention program on nurses' HIV-related knowledge and attitudes in Lithuania. The program focused on HIV epidemiology, transmission, coinfections, treatment and care, risk contacts, and moral and ethical dilemmas. It was designed to increase nurses' knowledge and positive attitudes concerning HIV. The study used a randomized controlled trial design with two experimental groups ([EG]; EG1, n = 63, EG2, n= 63) and one control group (n= 59) in three Lithuanian hospitals. Data were collected using a questionnaire developed by Held (1993). The questionnaire included questions about participant demographic characteristics, knowledge, and attitudes toward those living with HIV. The education intervention, which combined a 2-day workshop and written materials, had a positive effect on the knowledge levels of nurses. However, written materials alone failed to improve nurses' knowledge or change their attitudes. Copyright © 2011 Association of Nurses in AIDS Care. Published by Elsevier Inc. All rights reserved.
